# Trunk muscle activation during movement with a new exercise device for lumbo‐pelvic reconditioning

**DOI:** 10.14814/phy2.13188

**Published:** 2017-03-21

**Authors:** Tobias Weber, Dorothée Debuse, Sauro E. Salomoni, Edith L. Elgueta Cancino, Enrico De Martino, Nick Caplan, Volker Damann, Jonathan Scott, Paul W Hodges

**Affiliations:** ^1^ European Space AgencyEuropean Astronaut CentreSpace Medicine Office (HSO‐AM)CologneGermany; ^2^ KBRWyle GmbHCologneGermany; ^3^ Faculty of Health and Life SciencesNorthumbria UniversityNewcastle upon TyneUnited Kingdom; ^4^ The University of QueenslandNHMRC Centre of Clinical Research Excellence in Spinal Pain, Injury and HealthSchool of Health and Rehabilitation SciencesBrisbaneQueenslandAustralia; ^5^ Sports Medicine Specialisation School, Medicine, Surgery and Neurosciences DepartmentUniversity of SienaToscanaItaly

**Keywords:** Deep spinal muscles, exercise device, fine‐wire electromyography, lumbar spine, rehabilitation

## Abstract

Gravitational unloading leads to adaptations of the human body, including the spine and its adjacent structures, making it more vulnerable to injury and pain. The Functional Re‐adaptive Exercise Device (FRED) has been developed to activate the deep spinal muscles, *lumbar multifidus* (LM) and *transversus abdominis* (TrA), that provide inter‐segmental control and spinal protection. The FRED provides an unstable base of support and combines weight bearing in up‐right posture with side alternating, elliptical leg movements, without any resistance to movement. The present study investigated the activation of LM, TrA, obliquus externus (OE), obliquus internus (OI), abdominis, and erector spinae (ES) during FRED exercise using intramuscular fine‐wire and surface EMG. Nine healthy male volunteers (27 ± 5 years) have been recruited for the study. FRED exercise was compared with treadmill walking. It was confirmed that LM and TrA were continually active during FRED exercise. Compared with walking, FRED exercise resulted in similar mean activation of LM and TrA, less activation of OE, OI, ES, and greater variability of lumbo‐pelvic muscle activation patterns between individual FRED/gait cycles. These data suggest that FRED continuously engages LM and TrA, and therefore, has the potential as a stationary exercise device to train these muscles.

## Introduction

Absence of effects of gravity in Low Earth Orbit, reduces the magnitude and frequency of mechanical forces acting on the human body, resulting in profound bone loss in the lower limb and atrophy of some (in particular the so‐called “anti‐gravity”) muscles (Fitts et al. 2001; Chang et al. 2016). Astronauts also experience flattening of the spinal curvatures and lower back pain (LBP) in‐flight (Kerstman et al. 2012) and they are at increased risk of intervertebral disc (IVD) herniation on return to Earth (Johnston et al. 2010).

Long‐term bed‐rest (LTBR) is used as a ground‐based analog of microgravity, and has been found to induce similar changes, including: atrophy of deep spinal muscles, IVD swelling, and a reduced lordotic lumbar spine (Belavy et al. 2011; Hides et al. 2011b). Furthermore, spinal extensor muscle activation becomes more phasic in nature and this persists for at least 6 months following re‐ambulation (Belavy et al. 2007).

But also people with LBP on Earth display atrophy and altered recruitment of the deep spinal muscles (Hodges and Richardson 1996; MacDonald et al. 2009). Two deeply situated spinal muscles that make important contributions to spine control are commonly affected in LBP: the *transversus abdominis* (TrA) and *lumbar multifidus* (LM) muscles (Hodges 1999). Both contribute to inter‐segmental control of the spine and pelvis via extensive attachments to vertebrae and pelvic segments (Wilke et al. 1995; Hodges et al. 2003), and are activated in various up‐right movements, often in a manner that is tonic (sustained) and not specific to the direction of internal and external forces (Hodges and Richardson 1997; Moseley et al. 2002). The morphology and function of these muscles are related to spinal integrity and the development of LBP (Hodges and Richardson 1996; Belavy et al. 2011; Hides et al. 2011a), and individuals with LBP display differences in the morphology and behavior similar to those observed after gravitational unloading (i.e. reduced (Ferreira et al. 2004), delayed (Hodges and Richardson 1997; Moseley et al. 2002) and more phasic (Saunders et al. 2004a) activation). Therefore, it is an important aim of the state‐of‐the‐art exercise interventions to prevent or treat LBP to improve motor control of LM and TrA (Hodges et al. 2013c).

**Figure 1:**
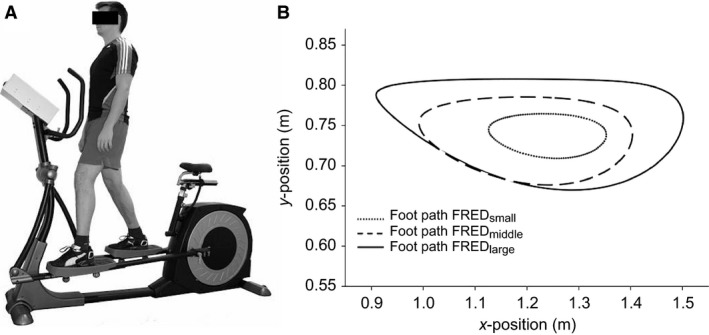
The FRED. (A) FRED device in use. (B) Foot paths are shown for the three amplitudes investigated in this study generated using a biomechanical model of the FRED (Lindenroth et al. 2015). The plot shows that the dimensions of the ellipses increase with increasing FRED amplitudes. FRED, Functional Re‐adaptive Exercise Device.

Several exercises (Hodges and Richardson 1996; Hodges 1999; Hides et al. 2011a; Hodges et al. 2013c) are known to activate TrA and LM, and change their recruitment patterns in terms of activation levels, timing, and interplay with other trunk muscles. (Tsao and Hodges 2007, 2008; Tsao et al. 2011; Hodges et al. 2013a). These exercises train activation of these muscles before their integration into function during habitual movements. That means that a currently used strategy to train theses muscles and treat LBP is to first teach patients how to activate them in isolation and then incrementally integrate the newly learned activation patterns into more complex‐, and finally into habitual everyday movements (e.g. reaching over head or standing up from a chair) (Hodges et al. 2013a). However, specific recruitment strategies such as learning how to activate certain trunk muscles in isolation and then to integrate isolated contractions into more complex movements, or how to de‐activate certain trunk muscles where disadvantageous over‐activity is present can be difficult to teach and learn, requiring supervision by a physiotherapist to confirm correct activation (Van et al. 2006; McPherson and Watson 2014). Availability of a simple approach could aid translation to practice. The *Functional Re‐adaptive Exercise Device* (FRED; Fig. 1) (Debuse et al. 2013; Caplan et al. 2015) was designed on the premise that alternating lower limb movement in an up‐right, weight‐bearing posture, combined with an unstable base of support, would encourage TrA and LM activation. B‐mode ultrasound and surface electromyography (sEMG) studies of FRED exercise provide data indicative of tonic activation of TrA and LM (Caplan et al. 2015), and with less pelvic and spinal motion than over‐ground walking (Gibbon et al. 2013). The device also induces greater activation of trunk extensor muscles and less activation of trunk flexor muscles than walking (Caplan et al. 2015). As these features are opposite to the changes observed following LTBR (Belavy et al. 2011) and microgravity (Hides et al. 2016; Chang et al. 2016) (personal communication, European Space Agency physiotherapist), FRED exercise might be used to help correct changes in trunk muscle activation following prolonged gravitational unloading (Evetts et al. 2014). Moreover, as the device appears to address muscular deficits proposed to play a role in the LBP (Hodges and Richardson 1996; Hides et al. 2011a), FRED exercise could also be useful for these patients.

Although estimates of muscle activation with FRED exercise from muscle thickness measures with ultrasound imaging (Debuse et al. 2013) and surface EMG (Caplan et al. 2015) are encouraging, both have limitations (e.g. cross‐talk between muscles for surface EMG; non‐linear relationship between muscle thickness and muscle activation for ultrasound imaging) for interpretation of activation of the deeply situated TrA and LM (Brown and McGill 2008). Considering the limitations of previous studies to investigate the FRED, the present study sought to illuminate the immediate effects more in‐depth using intramuscular fine‐wire EMG. The aims of this investigation were (1) to compare lumbopelvic muscle activation patterns during FRED exercise and treadmill walking, and (2) to assess the effect of different FRED amplitudes (as shown in Fig. 1) on lumbopelvic muscle activation.

## Methods

### Participants

Nine healthy male volunteers (mean [SD] age: 27 (5) years; height: 1.74 (0.05) m; mass: 72.8 (10.3) kg, body mass index: 24.1 [2.7]) with no history of LBP, or lower limb pain or injury participated in the study. The study was publicly advertised at the University of Queensland, however, only male volunteers responded to the announcement. The fact that only male volunteers could be recruited should not have compromised the findings and generalizability of results given that immediate trunk muscle activation was selected as the main outcome parameter and it is not known to be influenced by gender. Risks and procedures of the study were explained and all participants provided written, informed consent before participation. The study was approved by the Institutional Medical Research Ethics Committee and all procedures were in accordance with the Declaration of Helsinki.

### Instrumentation

#### Intramuscular electromyography

Before electrode placement, the overlying skin was sterilized (Persist Plus sterilization swab sticks, BD, Franklin Lakes). Intramuscular bipolar fine‐wire electrodes (two Teflon‐coated 75 *μ*m stainless‐steel wires with 1 mm insulation removed from the ends, bent back to form hooks at 2‐ and 3‐mm length, threaded into a hypodermic 0.50 × 70 or 0.50 × 32 mm‐needle) were inserted with B‐mode ultrasound guidance (Aixplorer, Supersonic Imagine, Aix‐en‐Provence, France) into the trunk muscles on the right‐hand side. Electrodes were positioned as follows:

1. TrA, OI, and OE: Midway between the anterior superior iliac spine (ASIS) and the ribcage at depths determined by ultrasound imaging;
2. LM: Between L4/L5, 30 mm laterally to spinous processes until the needle reached the most medial part of the L4 lamina;
3. ES: At L2, 40 mm lateral to the spinous process.

#### Surface electromyography

Before electrode placement, the skin was prepared using an abrasive paste (Nuprep, Weaver and Company, Aurora) and cleansed with an alcohol swab. Bipolar surface electrodes (Blue Sensor N, Ambu, Ballerup, Denmark) with an inter‐electrode distance of 22 mm were placed on the skin approximately in parallel with the muscle fibers as follows:

1. OI/TrAs: medial to the ASIS in a horizontal orientation;
2. OEs: one electrode on the distal aspect of the 9th rib and one medial to this at an angle of ˜45° from horizontal;
3. LMs: adjacent to the L5 spinous process at an angle of ˜15° from vertical.

A reference electrode was placed over the iliac crest. EMG signals were pre‐amplified 2000 times, band‐pass filtered between 20 and 1000 Hz (Neurolog, Digitimer, Welwyn Garden City, UK) and recorded at a sampling rate of 2000 Hz using a Power1401 data acquisition system and Spike2 software (Cambridge Electronic Design, Cambridge, UK).

### Familiarization

Participants were familiarized with exercise on FRED and walking on a motor‐driven treadmill (BH, Vitoria‐Gasteiz, Spain). The 10‐min of familiarization with exercise on FRED included the three amplitude settings (Fig. 1), starting with the smallest. The paths traced by the feet at the three different amplitudes have been reported previously based on a biomechanical model (Lindenroth et al. 2015). Participants were instructed to maintain their feet in contact with the footplates at all times, hold their upper body as still as possible in an up‐right posture and maintain a frequency of 0.42 revolutions per second with a constant angular velocity throughout each complete rotation. Visual feedback of frequency and angular velocity was provided on a screen in front of the participant. For familiarization with treadmill walking, participants initially walked at 0.83 m sec^−1^ and the speed was increased in 0.056 m sec^−1^ increments until they reported that they were walking at their estimated “natural” speed. After 5 min of walking at their natural speed, they walked for 5 min at a speed (0.75 m sec^−1^) and a stride rate of 0.42 Hz (two steps in one stride) that were matched to the FRED settings in its middle amplitude.

### Data collection

Participants completed five exercise conditions: FRED exercise at three different amplitudes (FRED_small_, FRED_middle_, FRED_large_), and treadmill walking at their natural speed (Gait_natural_) and that matched to FRED_middle_ (Gait_matched_). The order was randomized using a sequence generated by www.randomizer.org. Each exercise condition was performed for 90 sec, with the final 30 sec used for analysis. Between conditions, participants rested for 120 sec in a standardized standing position on the floor. During FRED exercise, a trigger signal was recorded from the internal rotary encoder (RP6010, ifm Electronic GmbH, Essen, Germany) to provide a marker for each completed cycle. For treadmill walking, a footswitch (0.5 inch force sensing resistor, Trossen Robotics) was worn under the heel of the right shoe insole to mark each heel‐strike.

### Signal processing

Data were processed off‐line using Matlab (Version 2014a, Mathworks, Natick, MA). For each exercise trial, the trigger signals were used to divide the final 30 sec into individual revolutions or gait cycles. EMG data were visually checked for movement artifacts and any revolutions/gait cycles that included artifacts were removed before further analysis. From a total of 360 recordings, 16 (LMs: 9; OE: 2; OI: 4: OIs: 1) were removed and missing values were replaced using the expectation maximization algorithm (Dempster et al. 1977). EMG data were high‐pass filtered to remove any minor residual artifacts (fine‐wire: 50 Hz; surface: 30 Hz), full‐wave rectified, smoothed using a moving average filter with a time constant of 100 msec, time normalized and averaged across individual cycles.

The processed signals were used to determine mean (EMG_mean_), peak (EMG_peak_), and minimum (EMG_min_) amplitudes of the averaged signal (averaged curve of all individual FRED/gait cycles). The time (percentage of each revolution/cycle) for which the muscle was active was calculated. The threshold for activation was defined as an EMG amplitude in excess of five SDs above mean baseline EMG (smallest EMG amplitude for 1 sec). The coefficient of variation between individual revolutions/cycles (Coeff_variation_) was calculated. The Coefficient of variation indicates how much the signal during each individual cycle is varying from all other cycles (bounded by 0 and 1; lower Coeff_variation_ values indicate greater variation between cycles). As the Coeff_variation_ were high (in particular during FRED exercise), mean, peak, and minimum EMG were also calculated for each separate FRED/gait cycle, before averaging all cycles (Cycle_mean_; Cycle_peak_; Cycle_min_, respectively). EMG amplitudes were normalized to the peak activation of the averaged signal (EMG_peak_) across all conditions as normalizing to EMG_peak_ appeared to be more reliable than normalizing to a maximum voluntary contraction (as it was initially planned), where a high inter‐participant variability was observed. Across all trunk muscles, highest EMG_peak_ values were observed during the gait conditions (typically during the stance phase), and it is thus a robust reference for normalization of amplitudes.

### Statistical analyses

After examining each variable for normality a repeated measures analysis of variance (ANOVA) was used to compare the five different conditions. When the main effect of *Condition* was significant (Greenhouse‐Geisser *P *<* *0.05), pairwise post‐hoc comparisons were undertaken using Fisher's least significant difference test (Fisher's LSD). Statistical analyses were performed using SPSS statistics software (Version 19, IBM, Armonk, New York). The results (*P*‐values) of all pairwise comparisons as well as the *P*‐values for the main effect *Condition* of the present statistical analysis are listed in Tables 1, 2.

**Table 1:** ANOVA and pairwise EMG comparisons of FRED exercise in the middle amplitude and treadmill walking

Measure		Muscle	Main effect (*P*)	Post‐hoc (*P*)
EMG_mean_	iEMG	TrA	0.296	—
		LM	0.118	—
		OI	0.032	FRED_middle_ < Gait_natural_; Gait_matched_–0.02; 0.01
		OE	0.006	FRED_middle_ < Gait_natural_; Gait_matched_–0.046; 0.019
		ES	0.001	FRED_middle_ < Gait_natural_; Gait_matched_ – 0.027; 0.027
	sEMG	LMs	0.01	FRED_middle_ > Gait_natural_–0.045
		OIs	0.476	—
		OEs	0.17	—
EMG_peak_	iEMG	TrA	0.107	—
		LM	0.001	FRED_middle_ < Gait_natural_; Gait_matched_–0.019; 0.024
		OI	0.006	FRED_middle_ < Gait_natural_; Gait_matched_–0.008; 0.004
		OE	0.02	FRED_middle_ < Gait_natural_; Gait_matched_–0.033; 0.018
		ES	<0.001	FRED_middle_ < Gait_natural_; Gait_matched_–<0.001; 0.002
	sEMG	LMs	0.001	FRED_middle_ < Gait_natural_–0.005
		OIs	0.056	—
		OEs	0.017	FRED_middle_ < Gait_matched_–0.05
EMG_min_	iEMG	TrA	0.012	—
		LM	0.023	FRED_middle_ > Gait_natural_; Gait_matched_–0.028; 0.038
		OI	0.3	—
		OE	0.55	—
		ES	0.2	—
	sEMG	LMs	0.001	FRED_middle_ > Gait_natural_; Gait_matched_–0.004; 0.006
		OIs	0.34	—
		OEs	0.26	—
Cycle_mean_	iEMG	TrA	0.18	—
		LM	0.23	—
		OI	0.083	—
		OE	0.006	FRED_middle_ < Gait_matched_–0.024
		ES	0.003	FRED_middle_ < Gait_natural_; Gait_matched_–0.037; 0.048
	sEMG	LMs	0.05	—
		OIs	0.4	—
		OEs	0.15	—
Cycle_peak_	iEMG	TrA	0.065	—
		LM	0.007	—
		OI	0.065	—
		OE	0.002	FRED_middle_ < Gait_natural_; Gait_matched_–0.014; 0.003
		ES	<0.001	FRED_middle_ < Gait_natural_; Gait_matched_–<0.001; 0.001
	sEMG	LMs	0.003	—
		OIs	0.115	—
		OEs	0.032	—
Cycle_min_	iEMG	TrA	0.044	—
		LM	0.29	—
		OI	0.7	—
		OE	0.19	—
		ES	0.26	—
	sEMG	LMs	0.003	FRED_middle_ > Gait_natural_; Gait_matched_–<0.005; 0.006
		OIs	0.67	—
		OEs	0.62	—
Coeff_variation_	iEMG	TrA	0.023	—
		LM	<0.001	FRED_middle_ < Gait_natural_; Gait_matched_–<0.001; <0.001
		OI	0.007	FRED_middle_ < Gait_natural_–0.041
		OE	0.003	FRED_middle_ < Gait_natural_; Gait_matched_–<0.001; 0.004
		ES	<0.001	FRED_middle_ < Gait_natural_; Gait_matched_ –<0.001; 0.002
	sEMG	LMs	<0.001	FRED_middle_ < Gait_natural_; Gait_matched_ –<0.001; 0.003
		OIs	0.007	—
		OEs	<0.001	FRED_middle_ < Gait_natural_; Gait_matched_–0.009; 0.004
Time active	iEMG	TrA	0.3	—
		LM	0.1	—
		OI	0.15	—
		OE	0.031	FRED_middle_ < Gait_natural_–0.024
		ES	0.08	—
	sEMG	LMs	0.2	—
		OIs	0.35	—
		OEs	0.62	—

Post‐hoc analyses were performed provided the *P*‐value for main effect (condition) was ≤ 0.05 while for pairwise comparisons only *P* ≤ 0.05 are presented.

IEMG, intramuscular EMG; sEMG, surface EMG.

**Table 2:** ANOVA and Pairwise EMG comparisons of FRED exercise in the three different amplitude settings

Measure		Muscle	Main effect (*P*)	Post‐hoc (*P*)
EMG_mean_	iEMG	TrA	0.296	—
		LM	0.118	—
		OI	0.032	FRED_middle_ < FRED_large_–0.012
		OE	0.006	FRED_small _< FRED_large_–0.037
		ES	0.001	FRED_small _< FRED_middle_; FRED_large_; FRED_middle_<FRED_large_–0.024; 0.005; 0.034
	sEMG	LMs	0.01	FRED_small _< FRED_middle_; FRED_large_ –0.005; 0.018
		OIs	0.476	—
		OEs	0.17	—
EMG_peak_	iEMG	TrA	0.107	—
		LM	0.001	FRED_small_ < FRED_middle_; FRED_large_ –0.008; 0.006
		OI	0.006	FRED_middle_ < FRED_large_–0.033
		OE	0.02	FRED_small_; FRED_middle_ < FRED_large_–0.012; 0.012
		ES	<0.001	FRED_small _< FRED_middle_; FRED_large_ –0.009; 0.006
	sEMG	LMs	0.001	FRED_small _< FRED_middle_; FRED_large_; FRED_middle_ <FRED_large_ –0.005; <0.001; 0.008
		OIs	0.056	—
		OEs	0.017	FRED_small_ < FRED_large_–0.002
EMG_min_	iEMG	TrA	0.012	—
		LM	0.023	—
		OI	0.3	—
		OE	0.55	—
		ES	0.2	—
	sEMG	LMs	0.001	FRED_small_ < FRED_middle_ –0.005
		OIs	0.34	—
		OEs	0.26	—
Cycle_mean_	iEMG	TrA	0.18	—
		LM	0.23	—
		OI	0.083	—
		OE	0.006	FRED_middle _< FRED_large_–0.038
		ES	0.003	FRED_small _< FRED_middle_; FRED_large_ –0.025; 0.011
	sEMG	LMs	0.05	FRED_small _< FRED_middle_; FRED_large_ –0.006; 0.017
		OIs	0.4	—
		OEs	0.15	—
Cycle_peak_	iEMG	TrA	0.065	—
		LM	0.007	FRED_small _<FRED_middle_; FRED_large_ –0.008; 0.01
		OI	0.065	—
		OE	0.002	FRED_small_; FRED_middle_ < FRED_large_–0.018; 0.023
		ES	<0.001	FRED_small _< FRED_middle_; FRED_large_; FRED_middle_ < FRED_large_ – 0.012; 0.001; 0.038
	sEMG	LMs	0.003	FRED_small _< FRED_middle_; FRED_large_; FRED_middle_ < FRED_large_–0.002; <0.001; 0.011
		OIs	0.115	—
		OEs	0.032	FRED_small _< FRED_large_ –0.003
Cycle_min_	iEMG	TrA	0.044	—
		LM	0.29	—
		OI	0.7	—
		OE	0.19	—
		ES	0.26	—
	sEMG	LMs	0.003	FRED_small _< FRED_middle_; FRED_large_ –0.001; 0.037
		OIs	0.67	—
		OEs	0.62	—
Coeff_variation_	iEMG	TrA	0.023	FRED_small _< FRED_large_ –0.025
		LM	<0.001	FRED_small _< FRED_middle_; FRED_large_–0.033; 0.006
		OI	0.007	—
		OE	0.003	—
		ES	<0.001	FRED_small _< FRED_middle_; FRED_large_–0.036; 0.009
	sEMG	LMs	<0.001	FRED_small_; FRED_middle_ < FRED_large_–0.01; 0.046
		OIs	0.007	FRED_small _< FRED_middle_; FRED_large_–0.031; 0.029
		OEs	<0.001	FRED_small _< FRED_middle_; FRED_large_ –0.015; 0.001
Time active	iEMG	TrA	0.3	—
		LM	0.1	—
		OI	0.15	—
		OE	0.031	FRED_middle_ < FRED_large_–0.025
		ES	0.08	—
	sEMG	LMs	0.2	—
		OIs	0.35	—
		OEs	0.62	—

Post‐hoc analyses were performed provided the *P* ‐value for main effect (condition) was ≤ 0.05 while for pairwise comparisons only *P* ≤ 0.05 are presented.

## Results

All participants completed the entire data collection with no adverse events.

### General features of EMG during FRED exercise and treadmill walking

**Figure 2:**
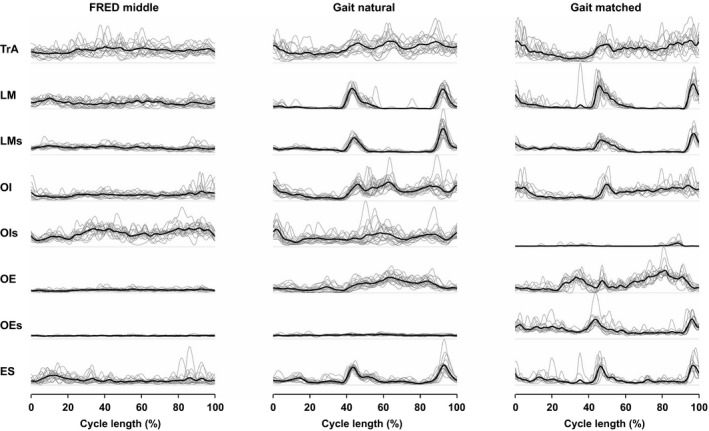
Representative processed EMG curves of one participant. Intramuscular and surface EMG recordings from one participant for FRED_middle_ and the two gait conditions. The thick black line depicts the averaged signal of all individual cycles (thin gray lines) as calculated analyzing the last 30 sec of each task. The light dotted line at the bottom of each plot indicates the zero reference for each channel. Cycle length represents one complete revolution on the FRED or the time from heel contact to heel contact of the right foot for treadmill walking. Note that unlike the gait data that begin and end with right foot strike, data for the FRED exercise are temporally organized to a set point in the smooth foot path.

Figure 2 depicts typical EMG recordings from one participant from the FRED_middle_ and the two treadmill conditions. Visual inspection of the signals reveals a high variability between individual cycles for FRED_middle._. This contrasts a more consistent pattern observed during treadmill walking. With treadmill walking, LM (LMs) and ES demonstrate typical phasic activation with bursts of activity aligned to heel‐strike, whereas activation during FRED_middle_ appears more random and not consistently aligned with any specific cycle event.

### Aim 1: Comparison between FRED exercise and treadmill walking

**Figure 3:**
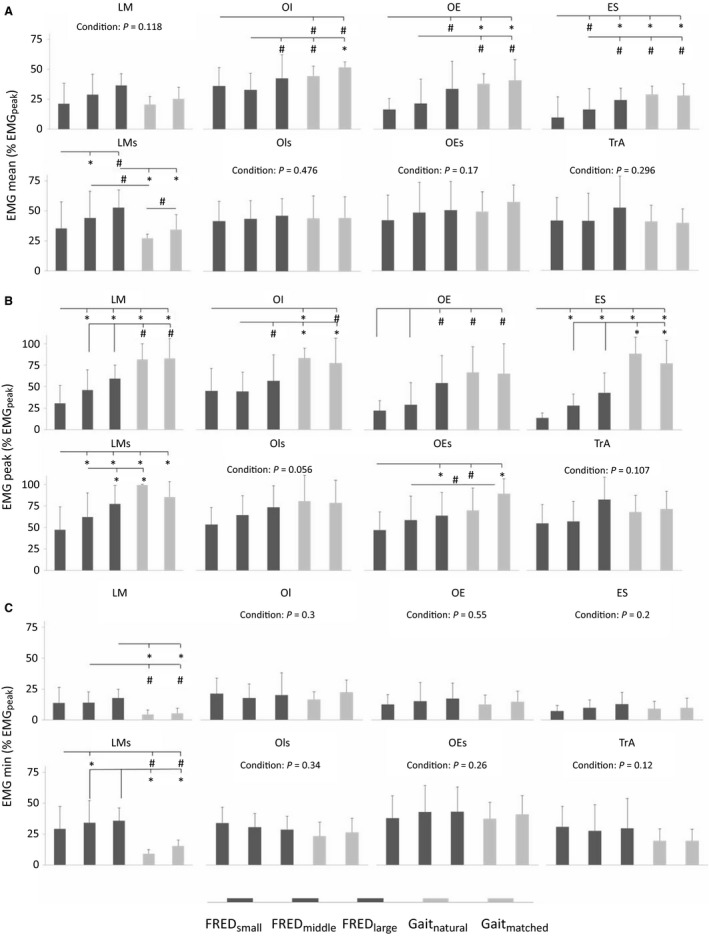
Mean, peak and min EMG amplitudes of the averaged EMG data. Group mean (SD) of intramuscular and surface EMG signals during the three FRED conditions (FRED_small_, FRED_middle_, FRED_large_) and treadmill walking at natural speed (Gait_natural_) and at a step frequency (0.84 Hz) matched to FRED_middle_ (Gait_matched_). The figure shows mean (A), peak (B) and minimum (C) amplitude of the averaged curves normalized to the greatest peak activation of the averaged signal for each muscle. Intramuscular EMG–LM, lumbar multifidus; OI, obliquus internus abdominis; OE, obliquus externus abdominis; ES, erector spinae; TrA, transversus abdominis; surface EMG‐LMs, lumbar multifidus; OIs, obliquus internus abdominis; OEs, obliquus externus abdominis. *P < 0.01 and ^#^ *P* < 0.05 for pairwise comparisons.

When data were averaged across consecutive cycles before analysis, recordings with fine‐wire electrodes revealed that EMG_mean_, EMG_peak_, and EMG_min_ for TrA showed no difference between FRED_middle_ and treadmill walking (Table 1, Fig. 3). LM EMG_mean_ was also not different when comparison was made between FRED_middle_ and walking, but LM EMG_peak_ was lower and EMG_min_ was observed higher during FRED_middle_ than walking, and these latter observations imply less fluctuation of activation (i.e. more “tonic”). Fine‐wire recordings of the superficial muscles OI, OE, and ES showed lower EMG_mean_ and EMG_peak_ during FRED_middle_ than both treadmill tasks (Table 1, Fig. 3), but EMG_min_ was not significantly different.

**Figure 4:**
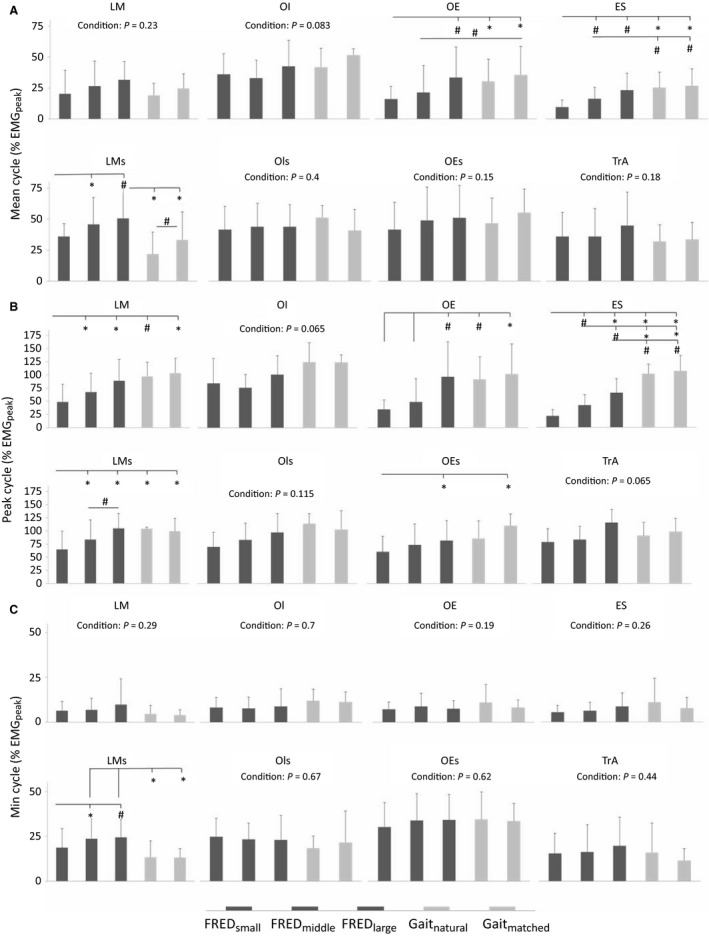
Mean, peak and min amplitudes determined from individual FRED/gait cycles. (A) Mean, (B) peak, and (C) minimum EMG recorded from all recorded intramuscular and surface EMG signals from individual FRED/gait cycles. EMG amplitudes were normalizd to the greatest peak activation of the averaged signal for each muscle. Intramuscular EMG–LM, lumbar multifidus; OI, obliquus internus abdominis; OE, obliquus externus abdominis; ES, erector spinae; TrA, transversus abdominis; surface EMG‐LMs, lumbar multifidus; OIs, obliquus internus abdominis; OEs, obliquus externus abdominis. *P < 0.01 and ^#^ *P* < 0.05 for pairwise comparisons.

Analysis of the data separately for each repetition, revealed similar observations to the analysis of the averaged EMG (Fig. 4). TrA and OI Cycle_mean_, Cycle_peak_, and Cycle_min_ did not differ between FRED_middle_ and treadmill walking. Although LM Cycle_mean_ and Cycle_min_ did not differ between FRED_middle_ and walking, LM Cycle_peak_ was lower in FRED_middle_ than both walking conditions. OE Cycle_mean_ was lower during FRED_middle_ than Gait_matched,_ OE Cycle_peak_ was less during FRED_middle_ than both walking tasks. OE Cycle_min_ did not differ between FRED_middle_ and the walking conditions. ES Cycle_mean_ and Cycle_peak_ during FRED_middle_ were lower than during both walking tasks, but ES Cycle_min_ did not differ between conditions.

The duration of activation (percentage of FRED/gait cycle) showed that OE was active for less time during FRED_middle_ than Gait_natural_. There were no difference for the other muscles.

**Figure 5:**
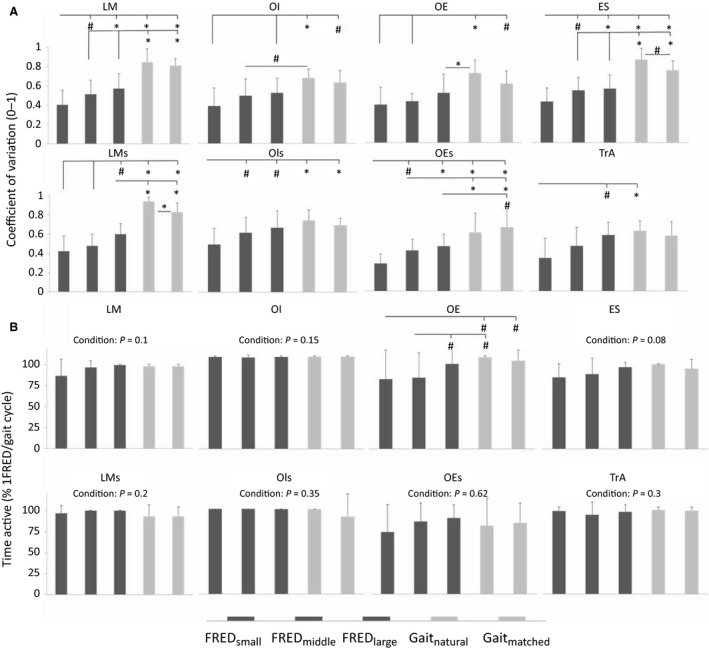
Coefficient of variation and time active. (A) The coefficient of variation indicates the variation of individual FRED/gait cycles from the averaged signal. (B) Time active indicates the percentage of time a muscle was active during the task. Intramuscular EMG–LM, lumbar multifidus; OI, obliquus internus abdominis; OE, obliquus externus abdominis; ES, erector spinae; TrA, transversus abdominis; surface EMG‐LMs, lumbar multifidus; OIs, obliquus internus abdominis; OEs, obliquus externus abdominis. *P < 0.01 and ^#^ *P* < 0.05 for pairwise comparisons.

The coefficient of variation between consecutive movement cycles was lower (i.e. more variable) for LM, OE, and ES during FRED_middle_ than both walking tasks, and OI Coeff_variation_ was lower during FRED_middle_ than Gait_natural_ only (Table 1, Fig. 5). The TrA Coeff_variation_ did not differ between FRED_middle_ and treadmill walking.

### Aim 2: Comparison between FRED exercise amplitudes

FRED exercise amplitude affected some aspects of trunk muscles activity. Although EMG_mean_ of LM and TrA were unaffected through amplitude changes of FRED, OE EMG_mean_ increased significantly from FRED_small_ to FRED_large_, and ES EMG_mean_ increased significantly from FRED_small_ to FRED_middle_, and from FRED_middle_ to FRED_large_. OI EMG_mean_ increased significantly from FRED_middle_ to FRED_large_.

TrA EMG_peak_ and EMG_min_ were not significantly different among the FRED conditions. LM EMG_peak_ increased from FRED_small_ to FRED_large_, whereas EMG_min_ of LM, OI, OE, ES were not different between conditions. OI EMG_peak_ was higher for FRED_large_ than FRED_middle._ OE EMG_peak_ increased significantly from FRED_small_ and FRED_middle_ to FRED_large_, and ES EMG_peak_ increased significantly from FRED_small_ to FRED_middle_ and FRED_large_.

Surface EMG recordings showed that LMs EMG_mean_ increased significantly from FRED_small_ to FRED_middle_ and from FRED_middle_ to FRED_large_, whereas OIs and OEs EMG_mean_ remained unaffected. LMs EMG_min_ increased significantly from FRED_small_ to FRED_middle_, whereas OIs and OEs EMG_min_ did not change. LMs EMG_peak_ increased significantly from FRED_small_ to FRED_middle_ and from FRED_middle_ to FRED_large_, OEs EMG_peak_ increased significantly from FRED_small_ to FRED_large_, whereas OIs EMG_peak_ remained unaffected (Table 2, Fig. 3). The duration of activation (percentage of FRED/gait cycle) did not differ between conditions for any muscle except OE, which was active for a longer period during FRED_large_ than FRED_middle_ (Table 2, Fig. 5).

Analysis of the data separately for each cycle were similar to the findings of the averaged data. Some minor differences were observed for OI, ES, and LMs (Table 2, Figs. 3, 4). Between‐cycle variation was affected by FRED amplitude. For intramuscular EMG recordings, LM, and ES Coeff_variation_ were lower for FRED_small_ than for FRED_middle_ and FRED_large_, and TrA Coeff_variation_ was lower for FRED_small_ than for FRED_large_ only (Fig. 5, Panel a). For surface EMG recordings, LMs Coeff_variation_ for FRED_small_ and FRED_middle_ exercise was lower than for FRED_large_, whereas OIs and OEs Coeff_variation_ were lower for FRED_small_ than for FRED_middle_ and FRED_large_ (Fig. 5).

## Discussion

This study presents novel results about the immediate effects of FRED exercise on lumbo‐pelvic muscle recruitment and adds important knowledge to the investigation process of a device that claims to be helpful in the recovery of LBP and in the rehabilitation phase after gravitational unloading (i.e. space flight, bed rest). Consistent with the proposed objective of FRED exercise, these results provide evidence that TrA and LM are activated continuously throughout cycles on the device. FRED exercise differed from treadmill in several respects, including more “tonic” pattern of activation of LM and lower activation of several superficial trunk muscles. These data highlight that FRED exercise may have therapeutic benefits for LBP patients and for individuals after prolonged gravitational unloading.

### Trunk muscle activity difference between FRED exercise and treadmill walking

Selective EMG recordings of trunk muscles with fine‐wire intramuscular electrodes during FRED exercise and treadmill walking revealed differences between these tasks, with some similarities and differences to previous non‐invasive recordings (Caplan et al. 2015). Previous studies of acute exercise with FRED reported the activation (surface EMG) of deep spinal muscles, greater trunk extensor muscle activation, less trunk flexor muscle activation, and a phasic‐to‐tonic shift of LM activation when compared with walking (Debuse et al. 2013; Caplan et al. 2015). Using selective fine‐wire recordings, the present data confirm sustained activation of LM and TrA during FRED exercise. Although no difference observed in the mean activation between FRED exercise and treadmill walking, consistent with the phasic‐to‐tonic shift in LM reported by Caplan et al. (2015), the pattern of intramuscular LM EMG during FRED exercise was characterized by less fluctuating continuous activation (greater minimum activation, lesser peak activation). This was observed for both surface and fine‐wire LM recordings in the present study.

The observation of less variation in LM EMG amplitude (lower peaks, greater minima) during FRED exercise than walking is likely to be explained by the absence of ground impacts at foot contact in FRED, which are known to lead to high peaks of LM activation in walking (Saunders et al. 2004b). It follows that there would be less difference in the pattern of TrA between FRED and walking as activation of that muscle is less dominated by peaks at foot contact in walking (Saunders et al. 2004b).

FRED exercise also aims to reduce the activation of more superficial trunk muscles that tend to have enhanced activation in the LBP (Hodges et al. 2013b). As reported from the surface EMG recordings (Caplan et al. 2015), mean trunk flexor (OI and OE) muscles activation was less in FRED exercise than over‐ground walking. In the present study, we also observed shorter duration of OE EMG bursts during FRED. Comparison of surface and fine‐wire recordings indicated that differences between tasks were more readily observed with selective fine‐wire electrodes, as surface recordings failed to show differences in some parameters. A departure from the observations of Caplan et al. (2015) is that trunk extensor activation (ES EMG) was less, rather than more during FRED. This difference is best explained by EMG cross‐talk, whereby each EMG recording site reflects the activation of multiple muscles within the recording field. Greater recording zone size for surface electrodes means those recordings will be more compromised by adjacent muscle activity. For ES, the previously used surface electrodes (Caplan et al. 2015) may have reflected activation of the superficially placed latissimus dorsi or thoroacolumbar erector spinae muscles, which we did not record. Similar to the argument presented for LM above, the lower mean and peak activation of the superficial muscles (OI, OE, and ES) during FRED is likely to be explained by removal of the high demand for trunk control related to foot strike.

A new observation was that activation of all trunk muscles was more variable between cycles (i.e. lower coefficient of variation) during FRED exercise. This contrasts the highly regular pattern of phasic modulation of activation of most muscles at consistent time points of each cycle in treadmill walking. There are several possible explanations. First, greater between‐cycle variation might reflect the novelty of this exercise, and participants' lack of familiarity. Analysis of habitual activities shows that motor units tend to fire more synchronously and more predictably when a movement is repeatedly performed (Enoka 1997). When quantified with the coefficient of variation (amount of variation of individual cycles from the mean of all cycles), a high value was observed for all trunk muscles during the walking trials. This is consistent with the highly familiar and repeatable nature of the task and associated muscle activity. Gait is a habitual movement for healthy humans that is at least in part controlled by spinal cord neural circuits (Bussel et al. 1996), and its interplay of muscle activation is genetically determined (Andersson et al. 2012) with fine‐tuning over decades of exposure.

Second, as mentioned above, FRED exercise lacks high ground reaction forces at foot strike. As activation of many of the trunk muscles is associated with foot strike (Saunders et al. 2004b) this would tend to constrain the variation between cycles, leading to a higher coefficient of variation.

Third, greater variation may reflect greater cycle‐to‐cycle variation in task demands. FRED exercise was designed to continuously challenge the muscles controlling lumbo‐pelvic posture and alignment. By making the base of support less stable, the intention was to enforce a need for the trunk muscles to continuously adjust the spine and pelvis position. This challenge is likely to vary between cycles, providing a potential explanation for less consistent EMG patterns. In the present study, the lowest correlation coefficient for all trunk muscles was observed during FRED exercise with the small or middle amplitude, indicating that the challenge may be greater (i.e. more unstable) in these situations.

### Changes in trunk muscle activation with FRED exercise amplitudes

Trunk muscle activation changed significantly when the foot‐path lengths during FRED exercise were altered through changes in the movement amplitudes. For most trunk muscles (LM, OI, OE/OEs, and ES) the greatest EMG_peak,_ EMG_mean_, and/or EMG_min_ activities were recorded during FRED exercise with the large amplitude, although the specific parameters differed between muscles. Two features of FRED exercise explain the increase with FRED amplitude. First, the large amplitude setting imposes greater excursion of the hips, placing greater demand for the control of proximal body segments. Second, the instability of the base of support is likely to be more difficult to control with large amplitudes. This will induce greater challenge for control, particularly for the participants in this study who were novice users (limited to 10 min of familiarization). During the large amplitude exercise it was not uncommon to observe “jerky” movements and associated peaks in trunk muscle activation. Lower cycle‐to‐cycle variation of LM/LMs, TrA, OI/OIs), ES and OEs with longer footpaths could imply that although this task is more challenging, the points in the task that were most challenging may be more consistent between repetitions which may tend to constrain the periods of most activity between repetitions.

### Potential role of FRED in rehabilitation of astronauts, individuals with LBP and following LTBR

Present results confirm that FRED exercise induces tonic activation of deeper trunk muscles, with lower mean activation of superficial spinal muscles (ES, OI, and OE) than treadmill walking at similar conditions. These features highlight the potential role of FRED exercise to counteract impaired (delayed and phasic) activation of deep lumbo‐pelvic muscles (Hodges and Richardson 1996; Ferreira et al. 2004; Saunders et al. 2004a; Hodges et al. 2013b) and increased activation of more superficial trunk muscles (van Dieen et al. 2003) observed in the LBP, as well as after LTBR (Belavy et al. 2007) and in the decreased size of deep spinal muscles as reported from astronauts after their missions (Hides et al. 2016; Chang et al. 2016).

Repeated exposure to postural perturbations can improve timing and amplitude of postural muscle activation (Horak and Nashner 1986). Further, repeated postural challenges in a specific environment developed new motor control strategies, which were transferrable to another environment (Horak and Nashner 1986). Taken together with our observed changes in muscle activation with FRED exercise, this implies FRED exercise could aid reversal of compromised neuromotor control and that the neuromotor control of trunk muscles trained through FRED exercise might be transferrable to other tasks. Clinical trials are needed to confirm the ability of FRED exercise to alter trunk muscle neuromotor control in the long term in individuals with deficits in trunk muscle function.

### Limitations

This study focused on a limited set of muscles based on the extensive literature highlighting compromised (LM and TrA) and augmented (OE, OI, and ES) activation in the LBP and after bed rest. However, this represents a subset of the trunk muscles that control the spine. Recent work highlights high variation between individuals (Hodges et al. 2013b) and involvement of additional muscles (*e.g. psoas*,* quadratus lumborum*) (Park et al. 2013). The present study shows differences (particularly for OI and OE) between surface and intramuscular recordings, which highlights that surface electrodes do not accurately represent their activation and highlights that fine‐wire electrodes are necessary to study the complex muscle system of the trunk.

Our interest in this study was to investigate individuals with no previous experience with FRED and a standardized period of familiarization (10 min) before data collection. It is unknown whether muscle activation patterns would differ with greater familiarity with FRED exercise, particularly when using the larger amplitudes.

## Conclusion

Intramuscular EMG recordings confirm that FRED exercise activates LM and TrA continuously. Moreover, compared with walking, trunk muscle activation during FRED exercise is associated with less activity of superficial muscles while the deep spinal muscles show similar mean activities. The patterns of activation during individual FRED cycles vary more than during walking. These data support the notion that FRED exercise might be effective to train the deep spinal muscles for populations where spinal muscle atrophy and compromised neuromotor control might be present (LBP, recovery after LTBR and space flight). Future studies are planned to investigate whether FRED exercise induces long‐term improvement in functional and morphological parameters of trunk muscles.

## Conflict of Interest

The authors have no conflicts of interests.
